# Supplementary material for: Cigarillo Flavor and Motivation to Quit among Co-Users of Cigarillos and Cannabis: A Structural Equation Modeling Approach
Source: Int J Environ Res Public Health. 2022 May 8;19(9):5727. doi: 10.3390/ijerph19095727 (PMC9101063; doi:10.3390/ijerph19095727)
Supplement: Supplementary file 1 [file ijerph-19-05727-s001.zip › ijerph-1685350-supplementary.pdf]

Supplemental Table S1

Table S1. Proposed Items for Measurement of Nicotine Dependence and Cigarillo Harm Perceptions.

**Nicotine Dependence**
**When I haven't been able to smoke for a few hours, the craving gets intolerable. (Never, Rarely, Sometimes, Often, Always)**
**I drop everything to go out and buy tobacco products. (Never, Rarely, Sometimes, Often, Always)**
I smoke before going into a situation where smoking is not allowed. (Never, Rarely, Sometimes, Often, Always)
**I find myself reaching for tobacco products without thinking about it. (Never, Rarely, Sometimes, Often, Always)**
**I chain smoke tobacco products. (Never, Rarely, Sometimes, Often, Always)**
**I feel anxious when I run out of tobacco products. (Never, Rarely, Sometimes, Often, Always)**
**The only thing that can calm me down is a tobacco product. (Never, Rarely, Sometimes, Often, Always)**
**I get irritated if I can't smoke a tobacco product when I feel like using one. (Never, Rarely, Sometimes, Often, Always)**
**I think about how I will get my next tobacco product. (Never, Rarely, Sometimes, Often, Always)**
I like to smoke an entire tobacco product by myself. (Never, Rarely, Sometimes, Often, Always)
When I am in a group, I prefer to smoke my tobacco product without sharing. (Never, Rarely, Sometimes, Often, Always)
I plan to have tobacco products available when I need them. (Never, Rarely, Sometimes, Often, Always)
**Perceived Cigarillo Harm**
**In general, how harmful do you think cigarillo smoking is to a person's health? (Not at all harmful, Somewhat harmful, Moderately harmful, Very harmful)**
In general, how harmful do you think blunt smoking is to a person's health? A blunt is a cigarillo that is filled with marijuana, cannabis, hash, THC, grass, pot, or weed. (Not at all harmful, Somewhat harmful, Moderately harmful, Very harmful)
**In general, how harmful do you think smoking a fruit flavored cigarillo is to a person's health? (Not at all harmful, Somewhat harmful, Moderately harmful, Very harmful)**

**In general, how harmful do you think smoking a sweet and candy flavored cigarillo is to a person’s health? (Not at all harmful, Somewhat harmful, Moderately harmful, Very harmful)**
**In general, how harmful do you think smoking a mint flavored cigarillo is to a person’s health? (Not at all harmful, Somewhat harmful, Moderately harmful, Very harmful)**
**In general, how harmful do you think smoking an alcohol flavored cigarillo is to a person’s health? (Not at all harmful, Somewhat harmful, Moderately harmful, Very harmful)**
**In general, how harmful do you think smoking a menthol flavored cigarillo is to a person’s health? (Not at all harmful, Somewhat harmful, Moderately harmful, Very harmful)**
**In general, how harmful do you think smoking a tobacco flavored cigarillo is to a person’s health? (Not at all harmful, Somewhat harmful, Moderately harmful, Very harmful)**
How addictive is flavored cigarillo smoking compared to cigarette smoking? (Much less addictive, Less addictive, They are the same, More addictive, Much more addictive)
How easy is it to quit flavored cigarillo smoking compared to cigarette smoking? (Much less addictive, Less addictive, They are the same, More addictive, Much more addictive)

Bolded items were included in the final model

Supplemental Table S2

Table S2. Structural Equation Model Fit Statistics by Multiply Imputed Dataset (n=5).

**Fit Statistic**	**Motivation to Quit Cigarillos**		**Motivation to Quit Cannabis**
	Overall	By Race/Ethnicity	
RMSEA (90% CI)			
Dataset 1	0.029 (0.012, 0.041)	0.023 (0.000, 0.038)	0.028 (0.012, 0.040)
Dataset 2	0.030 (0.014, 0.041)	0.033 (0.014, 0.046)	0.030 (0.014, 0.041)
Dataset 3	0.028 (0.011, 0.040)	0.019 (0.000, 0.036)	0.028 (0.010, 0.040)
Dataset 4	0.035 (0.022, 0.046)	0.034 (0.017, 0.047)	0.035 (0.022, 0.046)
Dataset 5	0.028 (0.012, 0.040)	0.020 (0.000, 0.037)	0.028 (0.012, 0.040)
CFI			
Dataset 1	0.992	0.995	0.992
Dataset 2	0.992	0.991	0.992
Dataset 3	0.992	0.996	0.992
Dataset 4	0.989	0.990	0.989
Dataset 5	0.992	0.996	0.992
TLI			
Dataset 1	0.991	0.995	0.991
Dataset 2	0.991	0.991	0.991
Dataset 3	0.991	0.996	0.991
Dataset 4	0.987	0.990	0.987
Dataset 5	0.991	0.996	0.991
SRMR			
Dataset 1	0.074	0.104	0.074
Dataset 2	0.073	0.096	0.073
Dataset 3	0.074	0.106	0.074
Dataset 4	0.075	0.100	0.075
Dataset 5	0.074	0.102	0.074

RMSEA=Root Mean Square Error of Approximation; CFI=Comparative Fit Index; TLI=Tucker Lewis Index; SRMR=standardized root mean squared residual

Supplemental Figure S1

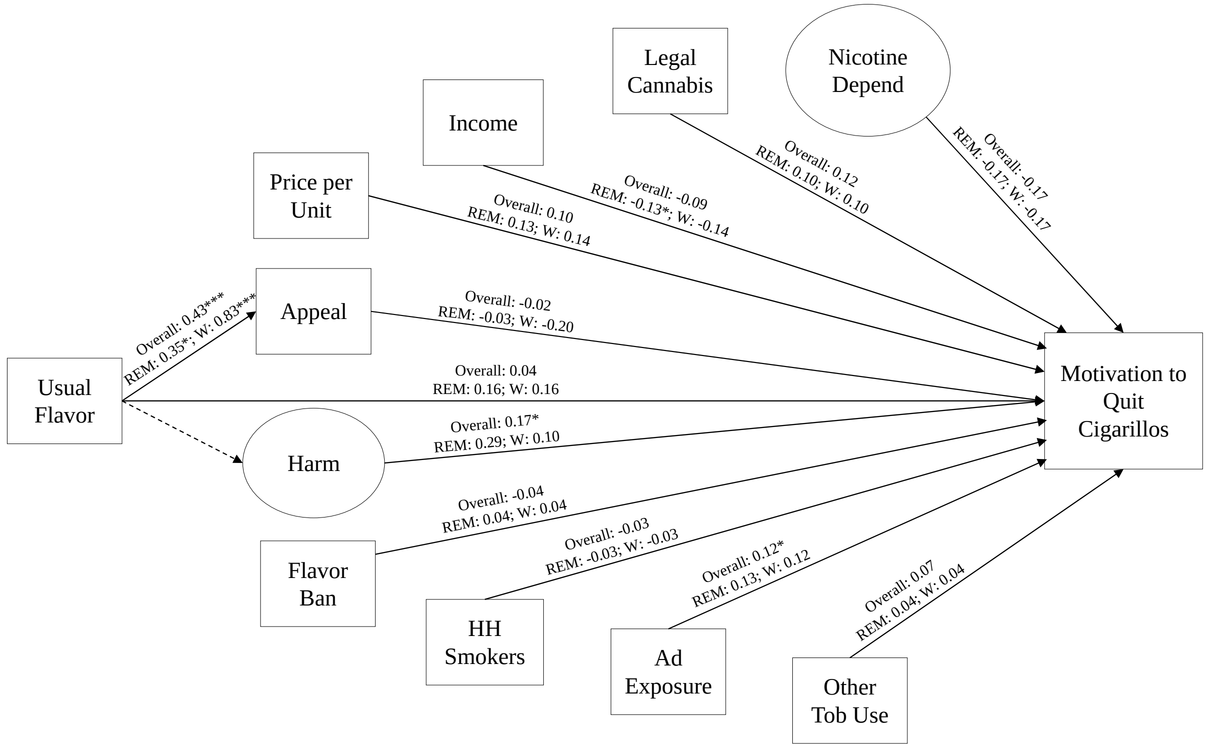

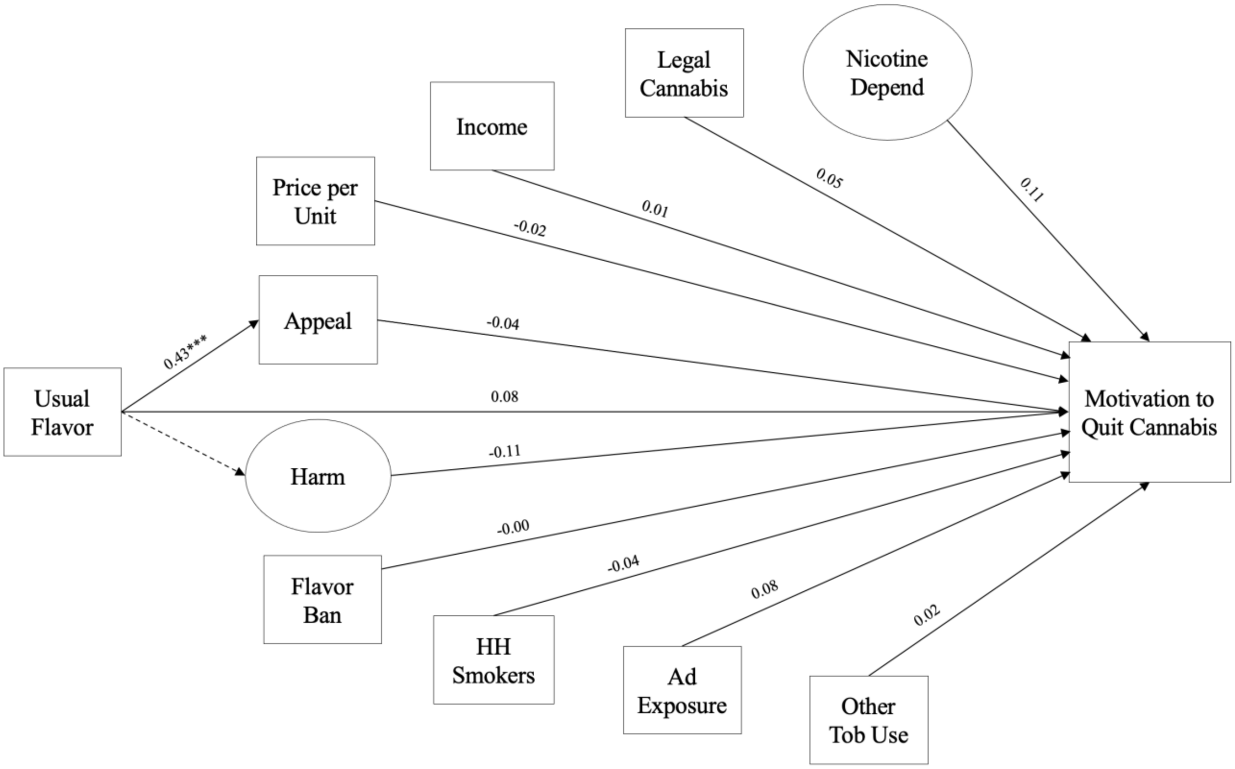

Figure S1. Final Structural Equation Model Results. *p<0.05; **p<0.01; ***p<0.001; HH=household, Ad=advertising, Tob=tobacco, Depend=dependence, REM=racial/ethnic minority, W=white
